# High prevalence of phenotypic pyrazinamide resistance and its association with *pncA* gene mutations in *Mycobacterium tuberculosis* isolates from Uganda

**DOI:** 10.1371/journal.pone.0232543

**Published:** 2020-05-15

**Authors:** Resty Naluyange, Gerald Mboowa, Kevin Komakech, Derrick Semugenze, David Patrick Kateete, Willy Ssengooba

**Affiliations:** 1 Department of Medical Microbiology, Mycobacteriology (BSL-3) Laboratory, Makerere University, Kampala, Uganda; 2 Department of Immunology and Molecular Biology, Makerere University, Kampala, Uganda; 3 The African Center of Excellence in Bioinformatics and Data Intensive Sciences, the Infectious Diseases Institute, McKinnell Knowledge Centre, Makerere University, Kampala, Uganda; 4 Makerere University Lung Institute, Makerere University College of Health Sciences, Kampala, Uganda; St Petersburg Pasteur Institute, RUSSIAN FEDERATION

## Abstract

**Introduction:**

Susceptibility testing for pyrazinamide (PZA), a cornerstone anti-TB drug is not commonly done in Uganda because it is expensive and characterized with technical difficulties thus resistance to this drug is less studied. Resistance is commonly associated with mutations in the *pncA* gene and its promoter region. However, these mutations vary geographically and those conferring phenotypic resistance are unknown in Uganda. This study determined the prevalence of PZA resistance and its association with *pncA* mutations.

**Materials and methods:**

Using a cross-sectional design, archived isolates collected during the Uganda national drug resistance survey between 2008–2011 were sub-cultured. PZA resistance was tested by BACTEC Mycobacterial Growth Indicator Tube (MGIT) 960 system. Sequence reads were downloaded from the NCBI Library and bioinformatics pipelines were used to screen for PZA resistance–conferring mutations.

**Results:**

The prevalence of phenotypic PZA resistance was found to be 21%. The sensitivity and specificity of *pncA* sequencing were 24% (95% CI, 9.36–45.13%) and 100% (73.54% - 100.0%) respectively. We identified four mutations associated with PZA phenotypic resistance in Uganda; K96R, T142R, R154G and V180F.

**Conclusion:**

There is a high prevalence of phenotypic PZA resistance among TB patients in Uganda. The low sensitivity of *pncA* gene sequencing confirms the already documented discordances suggesting other mechanisms of PZA resistance in *Mycobacterium tuberculosis*.

## Introduction

*Mycobacterium tuberculosis* (MTB), the causative agent of tuberculosis (TB), is a respiratory pathogen that causes significant morbidity and mortality especially in developing countries. *M*. *tuberculosis* has several strategies used to resist anti-microbial agents including the anti- TB drugs[1]. They have a highly hydrophobic cell wall that reduces permeability to many compounds[2]. Chromosomal mutations within the bacteria also affect the drug targets and inactivate the bacterial enzymes which in turn fail to activate the pro-drugs making them resistant[3]. PZA has been in use since 1952[4] though the frequencies and patterns of the resistance-conferring mutations are less studied in Uganda.

Resistance to PZA is associated with mutations in the *pncA* gene and its promoter [5] and recent research has reported the *rpsA* and *panD* genes[6]. However not all mutations that occur within these genes result into phenotypic resistance[7,8]. In regimens for MDR-TB treatment, PZA is mostly added without its susceptibility testing due to lack of a rapid and reliable phenotypic DST method. Phenotypic drug susceptibility testing (DST) by MGIT 960 is the gold standard for determining PZA resistance in TB and is recommended to investigate the susceptibility of the mycobacteria before administering anti-TB drugs[8,9]. *M*. *tuberculosis* H37Rv ATCC 27294 was used for PZA susceptible control while *M*. *bovis* BCG ATCC34540 was used as PZA–resistant control strains as recommended[10]. This study therefore determined the prevalence of phenotypic PZA resistance among the Uganda National TB drug resistance survey isolates and identified the mutations associated with PZA resistance. The mutations that are perfectly associated with phenotypic PZA resistance can be utilized in designing a molecular method for detection of PZA resistance. Molecular methods would present a more reliable method, with a shorter turnaround time and reduce the risks involved while manipulating live drug resistant strains involved in phenotypic DST.

## Materials and methods

### Design and setting

This was a cross- sectional study that used archived *Mycobacterium tuberculosis* isolates collected during the national drug resistance survey conducted between 2008–2011. The study population consisted of accessible and viable multidrug resistant, rifampicin and isoniazid mono-resistant and susceptible isolates obtained from the survey whose DST was determined using Lowenstein-Jensen proportional method. Susceptibility were tested at 40 mg/mL for rifampicin, 0.2 mg/mL for isoniazid and 10 mg/mL for streptomycin for which results were interpreted at week six, and 2 mg/mL for ethambutol for results were interpreted at week four [19]. The isolates were sub- cultured at the College of American Pathologists (CAP) Accredited, Mycobacteriology (BSL-3) Laboratory of the department of Medical Microbiology, Makerere University, Kampala, Uganda. Systematic sampling was used to randomly select isolates from the biorepository to get a representative sample of 307 as calculated by Taro Yamane (1967). Uncontaminated isolates from the survey were included in the study while the isolates in leaking or unlabeled vials and those missing from the freezers were excluded.

### Sub-culturing of the isolates

The isolates were retrieved from -80 ^0^C freezers and then thawed to recover the viable *Mycobacterium tuberculosis*. Selective media was made by adding amphotericin B, carbenicillin, polymyxin B, and trimethoprim lactate to minimize contamination. The bacterial isolates were aseptically inoculated in Middlebrook 7H9 selective broth for two weeks. After vortexing, 0.5 ml of the broth was transferred to Middlebrook 7H11 selective media plates and spread evenly. The plates were placed in a carbon dioxide incubator at 37°C. The plates were monitored for 6- weeks at a weekly interval for any mycobacterial growth and contamination. Completely contaminated plates were excluded from further experimentation, otherwise, they were re-decontaminated for recovery. For quality control, the isolates were accurately and rapidly reconfirmed as *Mycobacterium tuberculosis* using SD AgMPT64 kits as recommended[11].

### PZA susceptibility testing using BACTEC MGIT 960 system

Prior to the DST in BACTEC MGIT 960 system, for purity check, 0.1 μl of each organism suspension were streaked on sterile sheep blood agar plates and incubated at 37 ^O^C. Susceptibility testing was then done on pure isolates following the standard operating procedures. Briefly, each PZA drug vial was reconstituted with 2.5 ml of sterile distilled water to make a stock solution of 8000 μg/ml. Two 7 ml MGIT tubes labeled GC (Growth control) and PZA for each isolate were placed in their correct sequence for the 2-tube DST carrier as recommended in the BACTEC MGIT 960 user’s manual. The PZA tubes were inoculated by adding 0.5 μl of the organisms’ suspension and the contents mixed by gently inverting the tubes 4 times. The positive and negative control organisms were prepared as described for the isolates and separately added to the GC and PZA. The final PZA drug concentration was 100 μg/ml. All tubes were then entered into the BACTEC MGIT 960 machine for growth monitoring and interpretation.

### Genotypic PZA susceptibility testing by *pncA* sequencing

We downloaded sequences of isolates using purposive sampling were we obtained 37 isolates that were sequenced using Illumina TruSeq DNA technology in a previous study [7]. The sequences for each isolate were downloaded from NCBI Sequence read archive (SRA) and quality checked using FASTQC (https://www.bioinformatics.babraham.ac.uk/projects/fastqc/) software. Samples with good quality reads were further analyzed. Snippy tools[12] was used to call and annotate variants using *M*. *tuberculosi*s H37Rv as the reference genome. This tool uses the Burrow Wheeler Alignment tool (BWA)[13] to create SAM files for both the forward and reverse reads. Using the SAM tools[14], SAM files were converted to the Binary Alignment Map (BAM) files, then sorted and indexed. Variant calling was done using a haplotype-based variant caller FreeBayes software[15] with *Mycobacterium tuberculosis H37Rv* complete GenBank reference genome NCBI Reference Sequence[16] creating annotated variant call format (vcf) files. PZA resistance-conferring mutations were those categorized as so in the ReseqTB database (https://platform.reseqtb.org/). For samples that were PZA resistant but lacked the *pncA* mutations, customized bash scripts were used to explore mutations and polymorphisms within the genome that have been previously implicated in literature. Interpretation of PZA resistance was based on mutations reported in the TB Drug resistance Mutation Database and published literature[17,18].

### Statistical analysis

Socio-demographic and clinical characteristics were obtained from the previous study[19] and filled into an excel sheet (S1 Dataset). Data was analyzed in STATA V.13 where the frequencies for all Bactec 960 MGIT results, social demographics, and frequencies of various mutations and the corresponding number of isolates were coded. MEDCALC software was used to compute the Chi- square test and odds ratios to determine the associations between categorical variables. The frequencies of the mutations and polymorphisms in the *pncA* gene were recorded in a 2 X 2 table. Sensitivity and specificity of these mutations were computed using GraphPad software that uses the Fisher’s exact test.

### Ethical statement

The study was approved by the Higher Degrees Research and Ethics Committee (File no. SBS 540) of Makerere University, Kampala, Uganda—School of Biomedical Science, College of Health Sciences. The need for consent was waived by the ethics committee.

## Results

The study population consisted of isolates (n = 162), which were; mono-resistant to rifampicin 0.6% (n = 1), mono-resistant to isoniazid 11.3% (n = 18) while 11.3% (n = 18) were MDR (resistance to rifampicin and isoniazid). A total of 125 isolates were susceptible to all other drugs tested in the prevalence survey.

### Prevalence of phenotypic PZA resistance among the survey patients

Of the 162 isolates that were successfully cultured, 159 isolates had interpretable PZA susceptibility results. Of these, thirty-three were PZA resistant giving a prevalence of 20.8%.

### Distribution of *pncA* gene mutations among phenotypic PZA resistant isolates

Out of the 159 isolates that were investigated, 37 sequences were successfully downloaded. Of these, 25 were phenotypically resistant to pyrazinamide while 12 were susceptible. Six isolates had PZA resistance-conferring mutations in the *pncA* gene. One isolate had two mutations; V180F and T142R which confer phenotypic resistance. The most common mutation was V180F, and this occurred in four isolates. None of the PZA susceptible isolates harbored mutations in the *pnc*A and/or in its promoter region, Table 1.

**Table 1:** Frequencies of mutations in different regions in the *pnc*A gene.

Gene	Amino acid change	Phenotypic Susceptibility to PZA	Frequency
***pncA***	K96R	Resistant	1
	R154G	Resistant	1
	V180F +T142R	Resistant	1
	V180F	Resistant	3
	WT	Resistant	19
	WT	Sensitive	12

K- leucine, R- arginine, G- glycine, V- valine, F- phenylalanine, T- Threonine

WT: Wildtype

### Accuracy of *pncA* sequencing in detecting phenotypic PZA resistance

Of the 25 isolates with phenotypic susceptibility results, only six had PZA-resistance conferring mutations in the *pncA* gene and 19 isolates were wild type to PZA resistance conferring mutations in all known PZA resistance conferring genes. Thus resistance-conferring mutations in the *pncA* gene had a sensitivity of 24% (95% CI 9.36–45.13%) and a specificity of 100% (95% CI 73.54% - 100.00%), positive predictive value [PPV] 100.00% and negative predictive value [NPV] of 38.71% (31.92% - 65.60%), Table 2. The accuracy of *pncA* sequencing in predicting PZA was 48.65% (95% CI 31.92%- 65.60%). There were no mutations detected in the *panD* and the *rpsA* genes, Table 2.

**Table 2:** Showing diagnostic performance of PZA sequencing for PZA resistance determination.

Parameter	MGIT 960		Diagnostic accuracy			
*pncA* sequencing	Resistant	Susceptible	Sensitivity (%)	Specificity (%)	PPV (%)	NPV (%)
Mutation present	6	0	24 (9.36–45.13)	100.0 (73.54–100.0)	100	38.71
Mutation absent^s^	19	12				
Total	25	12				

R- Resistant, S–susceptible, PPV- positive predictive value, NPV—Negative predictive value

## Discussion

Our study using *M*. *tuberculosis* isolates from the national TB drug resistance survey shows high prevalence of phenotypic resistance to PZA. We further document low sensitivity but with high specificity of PZA resistance-conferring mutations among the phenotypically resistant strains. The standard phenotypic tests performed to detect resistance to PZA are slow and expensive which hinders timely management of TB. Additionally, there are several technical difficulties which are commonly associated with phenotypic testing consequently resulting into false diagnosis[20]. This is evident also in our results which show low sensitivity of *pncA* mutations that may be explained by alternative resistance mechanisms. Lack of knowledge about PZA resistance may increase the levels of resistance in Uganda due to transmission of the resistant strains that persist in the population. This suggests the need to globally document the circulating PZA resistance-conferring mutations to have a global catalogue of mutations that could provide a novel molecular test for PZA resistance. To the best of our knowledge, this is the first study to report prevalence of phenotypic PZA compared with genotypic resistance in Uganda.

PZA is an anti-TB drug used together with the other drugs to treat TB including those infected with MDR-TB. PZA being a cornerstone drug, a prevalence of 21% is quiet alarming. Inclusion of this drug in the treatment regimen without its susceptibility testing may amplify resistance and likely to cause unnecessary toxicity. The risk of mortality is nearly tripled when somebody with resistant PZA strains is put on a regimen containing this drug[21]. A study done in Kenya, showed a prevalence of 10.4%[22], whereas in the Republic of Peru, the prevalence of PZA resistance was about 7% indicating geographic diversity of the burden of PZA resistance.

This study did not detect any mutations in the *rpsA*, *panD* and *ClpC*1 genes. This report confirms four mutations in the *pncA* gene that are associated with phenotypic PZA resistance in Uganda, i.e. K96R, R154G, V180F and T142R which have also been reported in previous literature [23–25]. However, the study reported a low correlation between *pncA* gene mutations and phenotypic resistance. Nineteen resistant isolates had a wild type genotype implying that these would falsely be diagnosed as susceptible by molecular tests targeting mutations in the *pncA* gene. Our findings differ from the previous publication which suggested that phenotypic PZA resistance had a strong correlation with mutations in the coding and promoter regions of *pncA* gene[26]. However, our findings are consistent with other reports which reported more than 50% of PZA resistant isolates that lacked *pncA* gene mutations[27,28]. A study done in Brazil involving 97 clinical isolates identified 35 PZA resistant strains, 24 of which did not show PZAse activity[29]. Other studies done later showed that up to 30% of PZA resistant strains do not show any correlation between phenotypic PZA resistance and mutations in the *pncA* gene[18,29,30]. This imperfect correlation between genotypic and phenotypic DST methods for PZA suggests either other mechanisms of resistance or performance challenges mainly associated with the phenotypic DST methods. Nineteen (76%) of the phenotypically resistant isolates in our study had no resistance-conferring mutations. This suggests a need to design other studies to investigate other mechanisms of action of PZA that could explain the discordance results between the phenotypic and genotypic PZA resistance in Uganda.

However, the very high specificity of 100% implies that *pncA* sequencing clearly discriminates between those isolates that are susceptible from those that are resistant. In a similar study all the 18 phenotypically susceptible isolates had a wild type genotype[25]. Thus molecular-based methods such as whole genome sequencing holds great potential for the detection of PZA resistant TB as it also offers added value when compared with conventional susceptibility testing which is cumbersome [31] and time consuming. Pyrazinamide resistance remains largely unknown in the spectrum of drug resistant phenotypes [32]. However, the detection of resistance-conferring mutations and their inclusion in designing a molecular method for detection of PZA resistance may provide a reliable method of PZA susceptibility testing.

## Supporting information

S1 Dataset Laboratory information for pyrazinamide susceptibility testing for TB patients from the Uganda National drug resistance survey conducted between 2008–2011. (XLSX)
